# Surgical Management of a Canine Encephalocele Communicating with the Nasal Cavity

**DOI:** 10.3390/ani16091390

**Published:** 2026-05-02

**Authors:** Jin-Won Lee, Yongsun Kim, Hwi-Yool Kim

**Affiliations:** 1 Department of Veterinary Surgery, College of Veterinary Medicine, Konkuk University, Seoul 05025, Republic of Korea; kgo5341@naver.com; 2 Department of Veterinary Surgery, BON Animal Medical Center, Suwon 16305, Republic of Korea

**Keywords:** encephalocele, cribriform plate, dog, nasal cavity communication, skull base defect, meningoencephalocele

## Abstract

An encephalocele is a rare congenital or acquired cranial defect characterized by herniation of intracranial tissue through a defect in the skull base. In human and veterinary medicine, these lesions are frequently associated with abnormalities in neural tube development or structural weakness of the cranial bones, resulting in the protrusion of brain tissue and meninges through anatomical openings such as the cribriform plate. Although this condition has been extensively described in human neurosurgical research, reports on dogs remain limited, and the clinical significance of surgical intervention in cases with communication to the nasal cavity remains unclear. In this case, a young American Cocker Spaniel presented with seizures, prompting advanced diagnostic evaluation. Magnetic resonance imaging revealed a protrusion of the intracranial tissue through a defect in the cribriform plate extending into the nasal cavity. Surgical resection of the protruding tissue was performed, followed by skull base reconstruction. Histopathological examination demonstrated nervous tissue with chronic inflammatory changes without evidence of neoplasia. The patient recovered uneventfully after surgery and remained free of seizure recurrence during follow-up. Surgical management may represent a viable treatment option for seizure disorders in young dogs, particularly when persistent cranio-nasal communication is present, and provides a clinically relevant comparative model for similar cranial base defects described in human pathology.

## Simple Summary

Dogs occasionally develop rare abnormalities, in which the brain tissue protrudes through defects in the skull. These conditions are uncommon and may cause neurological symptoms such as seizures. As only a small number of cases have been reported in veterinary medicine, information regarding treatment and clinical outcomes remains limited. In this study, we describe the case of a young American Cocker Spaniel that developed seizures due to brain tissue extending through a defect in the skull into the nasal cavity. Advanced imaging revealed an abnormal connection between the brain and nasal cavity. Surgical removal of the protruding brain tissue and skull base reconstruction were performed. Following surgery, the dog recovered well, and no further seizures were observed during follow-up. This case highlights the importance of considering this rare condition when evaluating seizures in young dogs and demonstrates that surgical treatment may provide a favorable outcome in selected patients.

## 1. Introduction

An encephalocele refers to the herniation of intracranial contents through a defect in the skull, resulting in the protrusion of the meninges, and in some cases, brain parenchyma beyond the normal confines of the cranial vault [1]. In human medicine, encephaloceles are recognized as congenital anomalies and have been extensively described in the neurosurgical literature. Its incidence in humans has been estimated to range between approximately 1 in 3000 and 1 in 10,000 live births, although geographic variations in prevalence have been reported [2,3]. These lesions are commonly classified according to their anatomical location, with anterior skull base defects representing a distinct subgroup that frequently involves the frontoethmoidal or cribriform plate regions [4]. The underlying pathogenesis is generally attributed to abnormal neural tube closure or failure of mesodermal skull formation during early embryologic development [5,6].

In contrast to the relatively well-characterized presentation in humans, encephaloceles are rarely reported in veterinary patients. Only a limited number of cases have been described in dogs and cats, and their clinical significance remains incompletely understood [7,8,9]. The reported clinical manifestations in dogs vary widely and may include neurological abnormalities, such as seizures, behavioral changes, or progressive neurological dysfunction [8]. Recent reports and reviews have further emphasized that nasal encephaloceles and cribriform plate defects may present with variable neurological signs and may create clinically relevant communication between the sinonasal tract and the intracranial compartment [10,11] This anatomical relationship is important because persistent cranial–nasal communication can increase the risk of ascending contamination, chronic inflammation, and recurrent protrusion of intracranial tissue. For this reason, treatment planning should consider not only removal of the herniated tissue but also reliable reconstruction of the skull base barrier.

Owing to the limited number of veterinary cases reported, optimal treatment strategies remain poorly defined. Conservative management and surgical intervention have been reported, and therapeutic decision-making is typically guided by the presence of neurological signs, a risk of intracranial infection, and persistent communication between the intracranial structures and the sinonasal tract [12,13]. Surgical resection of the herniated neural tissue combined with reconstruction of the skull base has been reported in a small number of cases, with variable clinical outcomes [9,12].

The purpose of the present report is to describe the surgical management and clinical outcome of a young American Cocker Spaniel presenting with seizure activity associated with an encephalocele communicating with the nasal cavity, with particular emphasis on surgical decision-making and postoperative neurological stability.

## 2. Case Description

An 8-month-old spayed female American Cocker Spaniel presented with a history of seizures. According to the owner, the seizures developed recently, without prior neurological abnormalities. Physical examination findings were within normal limits. Neurological examination did not reveal abnormalities in cranial nerve function, spinal reflexes, or postural reactions. However, the history of seizure activity prompted further diagnostic evaluation [14].

### 2.1. MRI Findings

**Figure 1:**
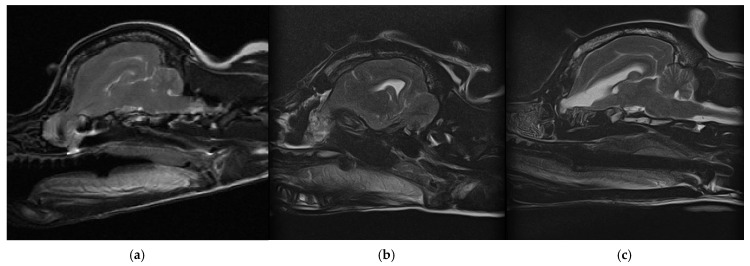
Preoperative and postoperative sagittal T2-weighted magnetic resonance images demonstrating the cranial–nasal interface and postoperative changes following surgical treatment. (**a**) Preoperative mid-sagittal image showing a soft-tissue structure extending from the intracranial compartment toward the nasal cavity through the cribriform plate region, with signal intensity similar to adjacent brain parenchyma. (**b**) An immediate postoperative sagittal image obtained after resection of the protruding tissue. (**c**) A follow-up sagittal image obtained approximately 1 year after surgery. No obvious intracranial tissue protrusion toward the nasal cavity is observed, and a thin linear hyperintense structure along the surgical margin may correspond to the reconstructed dural boundary.

Magnetic resonance imaging (MRI) of the brain revealed a protrusion of the brain parenchyma through a defect in the cribriform plate extending into the right nasal cavity. The lesion was located in the anterior cranial fossa and created apparent anatomical communication between the intracranial structures and nasal cavity. Cribriform plate-associated encephaloceles are rare causes of neurological signs in dogs and may increase the risk of ascending infection and chronic intracranial inflammation (Figure 1) [8,10].

### 2.2. Surgical Management

**Figure 2:**
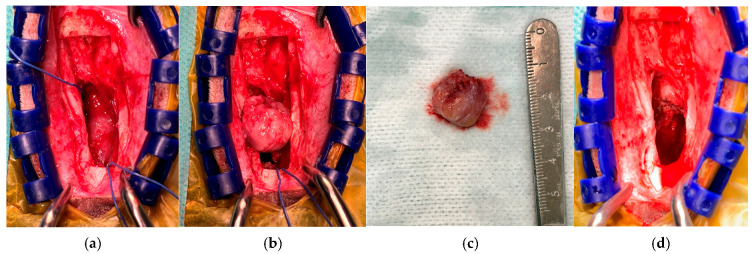
Intraoperative findings during surgical management of the encephalocele. (**a**) An intraoperative view following dorsal rhinotomy demonstrating brain tissue protruding through the cribriform plate into the nasal cavity. (**b**) An intraoperative view showing the herniated encephalocele tissue gently elevated from the surrounding structures prior to resection. (**c**) The excised tissue specimen obtained during surgery. The removed tissue measured approximately 1.4 cm in diameter. (**d**) The operative field after removal of the herniated tissue, with the skull base defect at the level of the cribriform plate visible. The defect was closed with an artificial dural substitute.

Surgical intervention was performed based on imaging findings and persistent neurological signs. Prior to surgery, as recorded in the medical record, phenobarbital (2 mg/kg, IV) was administered for seizure control. Anesthesia was induced using propofol (3 mg/kg, IV) in combination with midazolam (0.2 mg/kg, IV), and analgesia was provided with fentanyl (0.002 mg/kg, IV) along with the placement of a transdermal fentanyl patch (12 μg). After anesthetic stabilization, a dorsal rhinotomy approach was used to access the cranial–nasal communication site. After exposing the surgical field, the previously displaced bone flap was temporarily removed to provide access to the base of the skull. Herniated brain tissue protruding through the cribriform plate margin into the nasal cavity was carefully resected (Figure 2a).

Following the removal of the protruding tissue, a skull base defect at the level of the cribriform plate was identified, measuring approximately 10 mm × 10 mm. The excised tissue measured approximately 1.4 cm in diameter, which was larger than the defect size, likely reflecting expansion and deformation of the herniated tissue beyond the margins of the cranial opening. (Figure 2b). Reconstruction of the cranial base was performed using an artificial dural substitute (ReDura^®^, Medprin Biotech, Guangzhou, China), which was sutured to the surrounding native dura to restore separation between the intracranial and nasal compartments. The native dura surrounding the defect was friable and inflamed, making primary closure unreliable. The use of a synthetic graft allowed for stable sealing of the cranial–nasal interface and was intended to reduce the risk of cerebrospinal fluid leakage and ascending contamination.

Due to the incomplete preservation of the native bone margin, a polycaprolactone scaffold was used to cover the defect. The scaffold was placed over the dural substitute and secured using titanium micro-screws to restore structural continuity of the skull base. The surgical site was routinely closed.

### 2.3. Histological Findings

**Figure 3:**
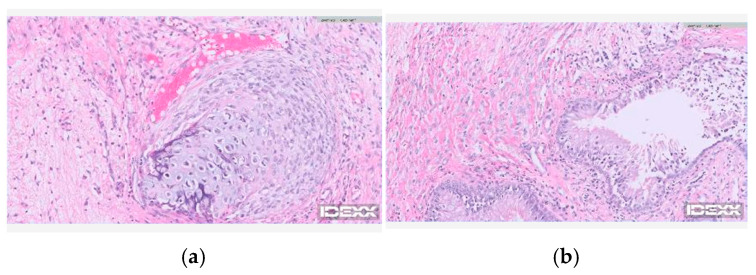
Histopathological findings of tissue resected from the cranial–nasal communication site. (**a**) Nervous tissue characterized by increased glial cell density and multifocal perivascular lymphocytic cuffs within the parenchyma. Multifocal aggregates of hemosiderin-laden macrophages are noted within perivascular spaces. (**b**) At the specimen margin, respiratory-type mucosa composed of a ciliated columnar epithelium with goblet cells forming glandular structures are identified. Adjacent stromal tissue shows reactive fibroplasia with focal cartilaginous metaplasia. Hematoxylin and eosin stain.

Histopathological examination revealed nervous tissue with chronic inflammatory changes. Multifocal perivascular lymphocytic cuffs and increased glial cell density were identified. Multifocal aggregates of hemosiderin-laden macrophages were also noted, suggesting a previous hemorrhage and edema. The respiratory epithelium with reactive fibroplasia and focal cartilage metaplasia was observed at the margin of the specimen, supporting the presence of an interface between the brain tissue and the nasal cavity (Figure 3).

### 2.4. Follow-Up

The patient recovered without immediate complications. Postoperative care included fluid therapy using a half-saline solution supplemented with vitamin B at a maintenance rate, together with fentanyl-based analgesia. Intravenous medications included cefotaxime (50 mg/kg), famotidine (0.5 mg/kg), tranexamic acid (10 mg/kg, BID), mannitol (0.5 g/kg, BID), and dexamethasone (0.2 mg/kg, SID). Vitamin K (1 mg/kg, SC) was also administered during the postoperative period. The patient was hospitalized for eight days following surgery and was discharged without neurological deficits. Follow-up magnetic resonance imaging performed immediately after surgery confirmed removal of the herniated brain tissue and restoration of the cranial base boundary. No additional seizure activity was observed during the clinical follow-up period, and no clinical signs were identified within the first two months after surgery. One-year follow-up imaging showed no evidence of recurrent intracranial tissue protrusion into the nasal cavity (Figure 1). The ventricular system appeared slightly more prominent in the rostral cerebral region compared with the immediate postoperative study.

## 3. Discussion

Encephaloceles are cranial defects through which intracranial tissues herniate beyond the normal boundaries of the skull and have been extensively described in human medicine [1,5]. In humans, these lesions commonly arise from developmental abnormalities that affect neural tube closure and cranial bone formation during embryogenesis [5,6]. Anterior skull base encephaloceles involving the frontoethmoidal or cribriform plate regions represent a recognized anatomical subtype and may result in communication between intracranial structures and the nasal cavity [4].

In contrast to the relatively well-characterized conditions in humans, encephaloceles have rarely been reported in veterinary patients. Only a small number of cases have been documented in dogs, and the clinical behavior of these lesions remains incompletely defined [7,8]. The reported clinical signs may include seizures, progressive neurological deficits, or incidental imaging findings, depending on the location and extent of the lesion [8].

In the present case, this anatomical feature, together with ongoing seizure activity, supported surgical intervention aimed at removing the herniated tissue and restoring a stable barrier between the intracranial and nasal compartments [15,16]. Although conservative management may be considered in selected cases, the persistence of cranio-nasal communication and the potential risk of intracranial complications were key factors in the decision to proceed with surgery. In the present case, the histopathological findings revealed chronic inflammatory features, including perivascular lymphocytic cuffs and reactive fibroplasia, which likely reflect prolonged irritation or inflammatory changes at the interface between the neural tissue and respiratory mucosa.

Surgical treatment of encephaloceles has been described in both human and veterinary research, typically involving resection of the herniated neural tissue followed by reconstruction of the skull base defect [12,13]. Surgical management of encephaloceles involving the cranial–nasal interface remains technically demanding, particularly when precise reconstruction of the skull base is required. In this case, the dorsal rhinotomy approach provided a direct and unobstructed surgical corridor to the ethmoidal–cribriform region, allowing for accurate identification of the defect and controlled reconstruction under direct visualization. Restoration of a durable barrier between the intracranial cavity and sinonasal tract is considered an essential component of surgical management to reduce the risk of infection and prevent recurrent herniation [13,17].

Choosing dorsal rhinotomy over conventional transfrontal craniotomy provided a more direct surgical corridor to the ethmoidal region. This approach minimized brain retraction and allowed for unobstructed visualization of the skull base defect, which was essential for precise reconstruction. Resection of the protruding neural tissue was followed by the reconstruction of the cribriform plate defect using a dural substitute sutured to the surrounding native dura. Replacement of the temporarily removed bone segment and stabilization with neuroplate fixation further restored the structural integrity of the skull base. In this case, the selection of the surgical approach was primarily dictated by the presence of herniated tissue within the nasal cavity. However, the ability to utilize a dorsal rhinotomy approach was facilitated by the anatomical configuration of the canine skull, which provides relatively direct access to the ethmoidal–cribriform region. This suggests that while lesion orientation determines the need for a sinonasal approach, species-specific anatomy influences its practical applicability. In human patients, anterior skull base encephaloceles are managed using a range of approaches, including endoscopic endonasal and transcranial techniques depending on anatomical constraints [18].

Postoperative imaging confirmed the absence of recurrent intracranial tissue protruding into the nasal cavity. The thin linear structure observed along the surgical margin on follow-up MRI may correspond to the reconstructed dural boundary created during surgical repair (Figure 1). While the reconstruction materials were not clearly visualized on follow-up MRI, this is likely due to the limited susceptibility artifact of titanium micro-screws and the signal characteristics of the polycaprolactone scaffold. The absence of recurrent protrusion and stable neurological status were considered the primary indicators of successful reconstruction. Mild prominence of the ventricular system was also observed in the follow-up study. However, the clinical significance of this imaging finding remains uncertain, as the patient remained neurologically stable and seizure-free during the observation period. In this case, the effectiveness of the reconstruction was supported by the absence of recurrent herniation and a stable neurological outcome over the follow-up period. However, potential limitations of the rhinotomy approach include sinonasal morbidity and the need for careful postoperative management. As this is a single case, the approach cannot be generalized but may be considered in selected cases where direct access is achievable.

Although encephaloceles may arise from congenital developmental abnormalities, traumatic or acquired etiologies have been described in the human and veterinary literature [1,19]. In the present case, the exact etiology could not be definitively determined based on the histopathological findings alone. Nevertheless, the favorable clinical outcome suggests that surgical treatment may be a reasonable therapeutic option for selected patients presenting with persistent cranial–nasal communication and neurological signs.

## 4. Conclusions

This report describes the successful surgical management of an encephalocele communicating with the nasal cavity in a young dog presenting with seizures. Surgical resection of the herniated neural tissue, combined with reconstruction of the cribriform plate defect, restored the separation between the intracranial and nasal compartments and was associated with favorable clinical outcomes.

Although encephaloceles are rare in veterinary medicine, this case highlights the importance of considering these conditions in the differential diagnoses of seizure disorders in young dogs. Surgical intervention may be an effective treatment option for persistent communication between the cranial and nasal cavities.

## Data Availability

All relevant clinical data supporting the findings of this report are included in this article. Additional information may be made available from the corresponding author upon reasonable request in accordance with privacy and ethical considerations.

## Abbreviations

The following abbreviations are used in this manuscript:

MRI	Magnetic resonance imaging
